# Comparison of Frequency and Severity of Sleep-Related Breathing Disorders in Children with Simple Obesity and Paediatric Patients with Prader–Willi Syndrome

**DOI:** 10.3390/jpm11020141

**Published:** 2021-02-18

**Authors:** Agnieszka Lecka-Ambroziak, Marta Wysocka-Mincewicz, Anna Świercz, Małgorzata Jędrzejczak, Mieczysław Szalecki

**Affiliations:** 1 Department of Endocrinology and Diabetology, The Children’s Memorial Health Institute, Al. Dzieci Polskich 20, 04-730 Warsaw, Poland; m.wysocka@ipczd.pl (M.W.-M.); a.swiercz@ipczd.pl (A.Ś.); m.szalecki@ipczd.pl (M.S.); 2 Centre of Diagnosis and Therapy of Respiratory Diseases Pul-Med, 25-322 Kielce, Poland; jedrzejczak1958@tlen.pl; 3 Collegium Medicum, Jan Kochanowski University, 25-369 Kielce, Poland

**Keywords:** simple obesity, Prader–Willi syndrome, sleep-related breathing disorders, obstructive sleep apnea, central sleep apnea

## Abstract

Sleep-related breathing disorders (SRBDs) can be present in children with simple obesity and with Prader–Willi syndrome (PWS) and influence an individual diagnostic and treatment approach. We compared frequency and severity of SRBDs in children with simple obesity and with PWS, both without and on recombinant human growth hormone (rhGH) treatment, and correlation of SRBDs with insulin resistance tests. A screening polysomnography-polygraphy (PSG), the oral glucose tolerance test (OGTT) and homeostasis model assessment of insulin resistance (HOMA-IR) were analysed in three groups of patients—with simple obesity (group 1, *n* = 30, mean age 14.2 years), patients with PWS without the rhGH therapy (group 2, *n* = 8, mean age 13.0 years) and during the rhGH treatment (group 3, *n* = 17, mean age 8.9 years). The oxygen desaturation index (ODI) was significantly higher in groups 2 and 3, compared to group 1 (*p* = 0.00), and hypopnea index (HI) was higher in group 1 (*p* = 0.03). Apnea–hypopnea index (AHI) and apnea index (AI) results positively correlated with the insulin resistance parameters in groups 1 and 3. The PSG values worsened along with the increasing insulin resistance in children with simple obesity and patients with PWS treated with rhGH that may lead to a change in the patients’ care.

## 1. Introduction

Sleep-related breathing disorders (SRBDs) have become a more recognized serious complication of obesity in paediatric population [1,2,3,4]. They can significantly worsen the proper psychomotor development in children as well as their physical and psychological health and quality of life [5]. 

Prader–Willi syndrome (PWS) is the common known genetic reason for obesity. It is a first recognized human genetic imprinting disorder, with the prevalence of 1 in 15,000 to 1 in 30,000, caused by lack of paternally-inherited genes on chromosome 15q11–q13. PWS results from either paternal deletion (DEL 15, 70%), maternal uniparental disomy (UPD 15, 15–30%) or imprinting centre defect (ID, 1%) [6]. 

The natural history of untreated patients with PWS leads from severe difficulties in feeding during the first year of life with failure to thrive, to lack of satiety developing from the early childhood. It results in uncontrolled development of obesity with increased rate of morbidity and mortality in untreated population of patients with PWS. The PWS is also characterized by typical dysmorphic features, hypotonia, psychomotor delay, short stature, hypogonadotrophic hypogonadism and intellectual and behavioural problems. The hypothalamic function disorders seem to be the main cause of the clinical symptoms and implicate the higher risk of central origin SRBD (central sleep apnea, CA). However, several other factors, like obesity, hypotonia, scoliosis, narrowing of upper airways, reduction of saliva excretion and adenoid/tonsillar hypertrophy, can also lead to obstructive sleep apnea (OSA) [6,7,8,9,10].

Recombinant human growth hormone (rhGH) treatment is a well-recognized therapy in patients with PWS, with a positive effect not only in the improvement of growth and final height, but mainly in body composition and muscle strength. Many observations conclude that this may positively influence obstructive breathing disorders [11,12,13,14].

SRBD can be assessed by polysomnography studies, including screening polysomnography and polygraphy. This procedure assesses respiratory flow, respiratory effort and blood oxygen saturation. Llombart et al. published the results of polygraphy studies in paediatric patients with tonsillar hypertrophy and SRBDs and in children with concomitant disease, which included three patients with PWS (one died when BiPAP (bi-level positive airway pressure) ventilation was stopped) [15]. 

Although there is a more common awareness of a possible high risk of SRBD in children with obesity, not all the previous studies prove these results. In the paediatric patients with PWS, both OSA and CA risk factors can be present. Moreover, both groups of children may present with similar metabolic complications, including insulin resistance.

Our research hypotheses include determining the specificity of SRBDs in patients with PWS and evaluating the impact of obesity and insulin resistance on the development of SRBDs in patients with simple obesity and PWS. In the groups of patients with PWS, we also aim to determine the influence of rhGH treatment on SRBDs. To investigate these questions we compare the frequency and severity of SRBDs, as well as the frequency of certain types of SRBDs in children with simple obesity and paediatric patients with PWS, both without and during the rhGH treatment, and we correlate SRBDs with insulin resistance tests.

## 2. Materials and Methods

We assessed the SRBDs in the Children’s Memorial Health Institute (CMHI) patients: Group 1—children with simple obesity (*n* = 30, mean age 14.2 years), group 2—patients with PWS without the rhGH therapy (*n* = 8, mean age 13.0 years), group 3—patients with PWS during the rhGH treatment (*n* = 17, mean age 8.9 years). The body mass index (BMI) was assessed according to the Polish BMI standard chart, the BMI SD was calculated using the LMS method (method to obtain SD, LMS parameters: Lambda for the skew, Mu for the median, and Sigma for the generalized coefficient of variation). 

The screening polysomnography-polygraphy (PSG) was performed with Porti 6 (groups 2 and 3) and Porti 7 (group 1) equipment (the difference in the model used was caused by a different time of the studies), assessing nasal respiratory flow, respiratory effort and blood oxygen saturation. The following indexes were evaluated: AHI, apnea index (AI) and hypopnea index (HI), with the mean time of apnea, the longest apnea and hypopnea; ODI and its specific parameters (the mean, the deepest and the longest desaturation); together with the mean saturation. The presence and severity of SRBDs, with the frequency of CA, OSA and mixed apneas, were determined. An experienced sleep specialist evaluated the PSG results according to the American Academy of Sleep Medicine (AASM) rules in each group of patients.

According to the AASM rules, the definitions of SRBDs in children are as follows: OSA lasts for at least 2 missed breaths, is associated with a >90% fall in the signal amplitude for ≥90% of the event and continued or increased inspiratory effort is present during the episode. CA is associated with absent inspiratory effort and lasts 2 missed breaths when associated with an arousal, an awakening or a ≥3% desaturation or at least 20 seconds without other conditions. Hypopnea is scored when the event lasts for at least 2 missed breaths, is associated with a >50% fall in the signal amplitude for ≥90% of the episode and is associated with an arousal, an awakening or a ≥3% desaturation. Oxygen desaturation index (ODI) is a number of ≥3% desaturation per hour [16]. 

The normative values of polysomnography in the paediatric population are also reported in a paper by Uliel in Chest 2004. The normal value for the apnea–hypopnea index (AHI) is <1/h. Moderate AHI is scored when the result is between 5 and 10/h and severe when AHI is more than 10/h. The desaturation value was scored as ≥4%, minimal normal O2 saturation as 92% [17]. However, the ODI norms differ in the studies in the paediatric groups of patients, with the desaturation value mainly scored as ≥3%. The range of normal ODI criteria in the paediatric population was also published as <2.6 desaturations of ≥4%/h [18,19,20,21,22].

Additionally, the insulin resistance was assessed with HOMA-IR (homeostasis model assessment of insulin resistance) and OGTT (oral glucose tolerance test) as a part of a routine clinical follow-up. The insulin levels were evaluated with a radioimmunoassay technique, as well as the insulin-like growth factor 1 (IGF1), that was monitored during the rhGH treatment in group 3. The OGTT was performed with the glucose dose of 1.75 g/kg (maximal dose 75 g). 

The study was approved by the Bioethics Committee of the Children’s Memorial Health Institute (7/KBE/2019, 20.03.2019). Written informed consent was obtained from the patients’ parents.

### Data Analysis

Statistical analyses were performed using statistical software Statistica version 6.0 Statsoft Company. Results are expressed as mean values and standard deviations (±SD). We compared the results between two groups at once. Parameters were evaluated with t-Student test for independent samples. Differences between the groups were tested by unpaired *t*-Student test or Mann–Whitney U test, as appropriate.

Correlations between the assessed parameters were evaluated with Pearson correlation and Spearman rank correlation dependently on distribution. *p* values < 0.05 were considered statistically significant.

ODI results of two patients from group 3 were technical unreliable and were excluded from the evaluation.

## 3. Results

Group 1: Patients with simple obesity, *n* = 30, 20 girls, 10 boys; 10 patients were treated with metformin for insulin resistance. The patients had no other concomitant diseases, there were no adenotonsillar hypertrophy cases. 

Group 2: Patients with PWS without the rhGH treatment, *n* = 8, four girls, four boys; the genetic diagnosis was DEL 15 in six, UPD 15 in one and abnormality in methylation pattern of *SNRPN* in one patient. One girl was treated with metformin after impaired glucose tolerance and insulin resistance were diagnosed in the OGTT. In one girl, the PSG was performed after rhGH therapy was interrupted due to obesity. Three patients had adenoid hypertrophy, confirmed in nasal endoscopy, one of them had a history of adenoidectomy. One patient experienced severe neck and chest burn a few years before enrolment in the study. There was a diagnosis of severe scoliosis in two patients, one of whom underwent three orthopaedic surgeries. 

Group 3: Patients with PWS during the rhGH therapy, *n* = 17, 7 girls, 10 boys; DEL 15 was diagnosed in 11, UPD 15 in two, abnormality in methylation pattern of *SNRPN* in three and an unbalanced chromosomal translocation between chromosome 15 and 21 with del 15q11–13 in one patient. The rhGH therapy was continued in most patients for greater than three years (mean time 4.0 ± 3.0years), with the mean GH dose 0.019 ± 0.006 mg/kg/day. The mean IGF1 values at the time of PSG were 385 ± 284.8 ng/ml, IGF1 SD 1.0 ± 0.9; in three patients IGF1 exceeded upper values of a normal range for age and sex (2.3–2.8 SD). 

Adenoid hypertrophy was diagnosed in four patients, three other patients underwent adenoidectomy eight months, nine months and three years before the PSG. One patient had a history of a soft palate plastic surgery a few years prior to the PSG. Significant scoliosis was diagnosed in six patients (one of them had mild cerebral palsy), one patient underwent two orthopaedic surgeries, two patients have been using spinal orthoses. 

The mean time between PSG and OGTT with HOMA-IR assessment in the patients with PWS was −0.2 ± 1.0 year.

The clinical characteristics and PSG results of the three groups are presented in Table 1 and Table 2.

The patients in group 3 were significantly younger than in group 1 and had lower BMI SD in comparison to the patients in groups 1 and 2 

There was a positive correlation between BMI SD and AHI with both AI and HI (*r* = 0.56, *p* = 0.001; *r* = 0.55, *p* = 0.002; *r* = 0.37, *p* = 0.04), OSA with mixed apneas (*r* = 0.45, *p* = 0.01; *r* = 0.49, *p* = 0.01) and ODI with desaturation <90% (*r* = 0.54, *p* = 0.002; *r* = 0.39, *p* = 0.03) in group 1. However, in group 3 we found a positive correlation between BMI SD and CA (*r* = 0.52, *p* = 0.04), with a tendency toward a positive correlation between BMI SD and AHI with AI and mixed apneas (*r* = 0.45, *p* = 0.068; *r* = 0.45, *p* = 0.067; *r* = 0.47, *p* = 0.059).

HI (hypopnea index) was higher in the children with simple obesity compared to the patients with PWS treated with rhGH (*p* = 0.03), but ODI with the deepest desaturation values were significantly higher in groups 2 and 3 compared to group 1 (*p* = 0.00). The mean desaturation was deeper and mixed apneas and the longest apnea results were higher, with a tendency towards a higher CA in group 3 (*p* = 0.11), and the longest hypopnea results were higher in group 2. 

The groups of patients with PWS presented with higher mixed apneas than the group with simple obesity, *p* = 0.01 for group 3 and *p* = 0.12 for group 2. The only statistical difference between the groups of patients with PWS was within HI, which was higher in group 2 (*p* = 0.02). However, there was also a trend towards higher AI and deeper mean desaturation in group 3 (*p* = 0.10 and *p* = 0.13, respectively). 

We also found similar differences when we analysed the PSG results in the groups of patients matched for age (in group 1—*n* = 13, seven girls, aged 13.0 ± 0.6; in group 3—*n* = 10, five girls, aged 12.1 ± 4.3 years; group 2 was analysed as a whole). The only exception was the lack of tendency towards deeper mean desaturation in group 3. 

The OGTT showed higher basal glucose and higher insulin levels at 120 min in group 1 compared to group 3, as well as higher glucose and insulin results at 120 min in group 2 compared to group 3. 

The HOMA-IR index was higher in group 1 compared to group 3 (*p* = 0.02), with no other statistical differences between the groups. Although the comparison of the OGTT results were similar when we checked them in the groups of patients matched for age (see above), we did not confirm the difference in the HOMA-IR results. The OGTT and HOMA-IR results are shown in Table 3.

There was a positive correlation between AHI with AI and insulin values at 0 min of the OGTT (*r* = 0.36, *p* = 0.047 and *r* = 0.44, *p* = 0.015) and AI and HOMA-IR (*r* = 0.40, *p* = 0.00), with a similar tendency regarding AHI (*r* = 0.35, *p* = 0.054) in group 1. OSA with mixed apneas also positively correlated with insulin values at 0 min of the OGTT and HOMA-IR.

In group 3, we found the correlation between AHI with AI and both glucose (*r* = 0.53, *p* = 0.03; *r* = 0.50, *p* = 0.04) and insulin values (*r* = 0.58, *p* = 0.02; *r* = 0.57, *p* = 0.02) at 120 min of the OGTT and between the mean desaturation, with the mean desaturation time and HOMA-IR (*r* = 0.65, *p* = 0.01; *r* = 0.54, *p* = 0.03).

## 4. Discussion

American Academy of Pediatrics (AAP) published a clinical practice guideline regarding obstructive sleep apnea syndrome (OSAS) in children in 2012 [4]. Although the guideline does not include patients with central apnea and hypoventilation syndromes, it states that OSAS is associated with certain risk factors that include adenotonsillar hypertrophy, obesity, craniofacial anomalies and neuromuscular disorders, all present in patients with PWS. 

The increase in obesity among the paediatric population led to more detailed sleep studies in this group of patients. Most of the authors conclude that SRBDs are more frequent in the obese paediatric group [3,4]. However, some of the papers do not prove this result, especially regarding the obstructive type of SRBDs [23]. We documented SRBDs in all 30 patients with simple obesity, with moderate AHI in nine and severe in 20 children, with the majority of obstructive types of SRBDs. Our results are similar to other studies comparing the SRBDs frequency in paediatric patients with obesity without other concomitant diseases, to healthy children, that show higher AHI with higher incidence of OSA [1,24]. In the research by Gachelin et al. regarding 102 children with morbid obesity (BMI 4.52 ± 1.5 SD), eight patients with severe OSA required ventilation support. These patients were characterised by a rapid mass increase in early childhood [2]. 

In comparison, in the patients with PWS, the SRBDs origin is more complex. There have been researches studying possible candidate genes for the sleep disturbances in patients with PWS, such as the paternally expressed *Snord116* gene (small nucleolar ribonucleic acid-116) prevalently expressed in the brain [25]. OSAs are not only determined by obesity in children with PWS not treated with rhGH, but also by different factors typical for the syndrome, such as hypotonia, scoliosis, narrowing of upper airways and reduction of saliva excretion. The additional hypothesis regards the treatment with rhGH. The therapy can lead to the increase of IGF1 and may affect the lymphoid tissue, causing the adenoid/tonsillar hypertrophy [13]. Moreover, CAs seem to be part of the syndrome and are most probably caused by hypothalamic dysfunction.

Most of our patients with PWS, regardless of the rhGH treatment, presented moderate to severe AHI. Only three patients, all treated with rhGH, showed mild AHI. Half of groups 2 and 3 had the severe AHI score. Interestingly, in both groups the high index of obstructive SRBD was found, similar to the children with simple obesity. In the PWS groups we documented the additional risk factors among those listed above, such as adenoid hypertrophy or scoliosis. We also observed the increased IGF1 levels during the rhGH treatment in the individual patients.

The tendency toward higher CA in group 3, and higher mixed apneas in both groups of children with PWS, is consistent with the observation of the hypothalamic origin of a part of SRBD in patients with PWS and seems to not improve with rhGH therapy.

In a paper by Cohen et al., CAs were more frequent in children with PWS for less than two years of life, and some of them were efficiently treated with supplemental oxygen [8]. Pavone et al. published an important multicentre study of 88 patients with PWS before rhGH therapy, showing a high incidence of SRBD in polygraphy studies and the need of upper airway surgery in nine and non-invasive support ventilation in 16 of the patients [7].

The insulin resistance index, HOMA-IR, was correlated with AI and OSA in group 1. In group 3, the positive correlation between HOMA-IR and the mean desaturation, with the mean desaturation time, may indicate worsening of the PSG parameters along with the worsening of the insulin sensitivity in the patients with PWS, even when treated with rhGH. It would be worthwhile to take this into consideration when deciding about metformin treatment for insulin resistance in these particular groups of patients. Moreover, there was an expected positive correlation between the BMI SD, AHI and OSA in group 1, but in group 3 we found a positive correlation between BMI SD and CA. This may suggest that an increase of BMI SD, even within the normal range, in the PWS group may worsen the CA, in spite of rhGH therapy. However, group 3 was relatively small, thus these results should be confirmed in a larger group of patients. We could not correlate the BMI SD and insulin resistance results with PSG values in group 2, as the number of PWS patients not treated with rhGH is not sufficient for the statistical analysis. 

In Sleep 2011, Wise et al. concluded that the PSG is not sufficient to predict death risk or monitor cardiorespiratory abnormalities in children [18]. However, the population of patients with PWS has many specific risk factors for developing SRBDs and; therefore, close attention to SRBDs is recommended, especially before and after the commencement of rhGH therapy [26]. This includes monitoring of any changes in breathing, particularly in sleep, and evaluation by oximetry or polysomnography, together with otorhinolaryngology assessment, within the first three to six months of rhGH treatment (consensus guidelines for GH therapy in patients with PWS, JCEM 2013) [27]. The longer rhGH therapy seems to improve the respiratory muscle function and; therefore, the OSA type of breathing disorders [11,27]. 

There are data regarding the possibility of cognition and behaviour impairment in children with OSAS [4,5]. The studies related to the influence of SRBD in the population of patients with PWS on psychomotor development, cognition and behaviour are not explicit [28,29,30]. The results show that SRBDs are only one of the factors influencing the cognition and behaviour in children with PWS. Nonetheless, the improvement in SRBDs probably leads to the better daily performance of these patients.

## 5. Conclusions

Our study proves the high incidence of SRBDs in obese children, both with simple obesity and in the group of patients with PWS without rhGH treatment. However, also patients with PWS on the rhGH therapy, without obesity, showed significant SRBDs. The severe AHI score was documented in two-thirds of group 1 and in half of both groups of patients with PWS, with higher ODI in children with PWS, regardless of rhGH therapy, and higher mixed apneas while on rhGH treatment in comparison to the group of patients with simple obesity. 

The main PSG values worsen along with the increasing insulin resistance in both children with simple obesity and patients with PWS treated with rhGH.

The high SRBDs frequency, within the groups of children representing risk factors of SRBDs, show the need for further investigations towards its exact effects on psychomotor development and daily performance. 

Therefore, the sleep studies in these groups of paediatric patients may lead to a change in an individual care approach.

## Figures and Tables

**Table 1:** The clinical characteristics (the mean value ± standard deviation, SD).

	Group 1	Group 2	Group 3
Number of patients	30	8	17
Age (years)	14.2 ± 1.3 *	13.0 ± 4.7	8.9 ± 5.1 *
BMI	31.9 ± 5.4 **	32.8 ± 6.0 **	18.3 ± 4.0 **
BMI SD	2 3 ± 0.5 **	2.4 ± 0 9 **	0.4 ± 1.1 **

BMI—body mass index. Group 1: Patients with simple obesity; group 2: PWS patients without the rhGH treatment; group 3: PWS patients during the rhGH therapy. * *p* < 0.05: Group 1 vs. 3, ** *p* < 0.05: Group 1 vs. 3, 2 vs. 3.

**Table 2:** The PSG indexes (the mean value ± standard deviation, SD).

PSG Indexes	Group 1	Group 2	Group 3
AHI	14.3 ± 12.7	10.9 ± 5.3	12.6 ± 8.2
AI	8.3 ± 9.9	4.9 ± 2.2	9.4 ± 7.1
HI	6.0 ± 4.7 *	6.0 ± 3.5 *	3.2 ± 1.9 *
CA	18.1 ± 27.1	18.8 ± 19.5	32.1 ± 29.4
OSA	37.3 ± 39.1	24 ± 7.2	30.6 ± 20.1
Mixed apneas	3.6 ± 10.9 **	10.3 ± 8.5	12.9 ± 13.8 **
The mean time of apnea (s)	11.2 ± 1.7	12.1 ± 2.6	12 ± 1.9
The longest apnea (s)	21.2 ± 7.9	29.4 ± 18.3	27.9 ± 8.6
The longest hypopnea (s)	27.3 ± 10.4	41.9 ± 19.0	32.7 ± 16.1
ODI	7.3 ± 7.4 ***	22.0 ± 13.0 ***	25.1 ± 17.4 ***
The mean desaturation (%)	92.0 ± 2.0	91.4 ± 1.8	89.3 ± 3.6
The deepest desaturation (%)	80.8 ± 7.2 ***	66.6 ± 3.5 ***	65 ± 7.3 ***
The longest desaturation (min)	1.1 ± 0.5	1.4 ± 0.6	1.3 ± 0.4
The mean saturation (%)	92.5 ± 11.8	93.1 ± 2.2	90.3 ± 6.4

PSG—screening polysomnography-polygraphy, AHI—apnea–hypopnea index, AI—apnea index, HI—hypopnea index, CA—central sleep apnea, OSA—obstructive sleep apnea, ODI—oxygen desaturation index. Group 1: Patients with simple obesity; group 2: PWS patients without the rhGH treatment; group 3: PWS patients during the rhGH therapy. * *p* < 0.05: Group 1 vs. 3, 2 vs. 3, ** *p* < 0.05: Group 1 vs. 3, *** *p* < 0.05: Group 1 vs. 2, 1 vs. 3. AHI norm = < 1/h, moderate score = 5–10/h, severe score = >10/h; ODI norm = < 2.6, desaturations of ≥ 4%/h.

**Table 3:** The insulin resistance assessment: Insulin and glucose in the 0’–120’ of the OGTT and HOMA-IR (the mean value ± standard deviation, SD).

Results	Group 1	Group 2	Group 3
HOMA IR	3.6 ± 1.7 *	2.7 ± 0.9	2.3 ± 2.2 *
Glucose 0’ (mg/dL)	90.6 ± 17.9 *	85.1 ± 5.5	76.2 ± 13.8 *
Glucose 120’ (mg/dL)	130.9 ± 40.4	140.4 ± 31.1 **	113.3 ± 20.1 **
Insulin 0’ (uIU/mL)	16.2 ± 7.9	12.8 ± 4.1	11.2 ± 9.2
Insulin 120’(uIU/mL)	117.7 ± 63.0 ***	74.3 ± 36.4 ***	34.2 ± 27.9 ***

OGTT—oral glucose tolerance test, HOMA-IR—homeostasis model assessment of insulin resistance. Group 1: Patients with simple obesity; group 2: PWS patients without the rhGH treatment; group 3: PWS patients during the rhGH therapy. * *p* < 0.05: Group 1 vs. 3, ** *p* < 0.05: Group 2 vs. 3, *** *p* < 0.05: Group 1 vs. 3, 2 vs. 3.

## Data Availability

The data presented in this study are available on request from the corresponding author. The data are not publicly available due to ethical restrictions.
